# In Silico Identification and Structural Characterization of High-Risk Missense SNVs in the Human *IL23R* Gene Relevant to Inflammatory Bowel Disease

**DOI:** 10.3390/genes17060699

**Published:** 2026-06-16

**Authors:** Gamze Altintas Kazar

**Affiliations:** Department of Biology, Faculty of Science, Trakya University, Edirne 22030, Turkey; gamzealtintas@trakya.edu.tr

**Keywords:** IL23R, inflammatory bowel disease, missense variant, in silico analysis, protein stability, single-nucleotide polymorphism

## Abstract

Background/Objectives: *IL23R* encodes a pivotal component of the IL-23/Th17 signaling axis and represents a validated genetic susceptibility locus for inflammatory bowel disease (IBD), psoriasis, and ankylosing spondylitis. Despite extensive GWAS data, the functional consequences of the full spectrum of *IL23R* missense single-nucleotide variants (SNVs) have not been systematically characterized. This study aimed to identify high-risk missense SNVs through a multi-tool in silico pipeline. Methods: A total of 723 missense SNVs from NCBI dbSNP were verified against transcript NM_144701.3/Q5VWK5-1 (629 aa) using Ensembl VEP (GRCh38). Sequential filtering was performed using applied SIFT, PolyPhen-2, PROVEAN, E-SNPs&GO, MutPred2, and ConSurf (grade ≥ 7); AlphaMissense and FATHMM-MKL were used as independent annotation layers. Protein stability was assessed with MuPro and DynaMut2 (AlphaFold2 AF-Q5VWK5-F1-v6; pLDDT = 68.19); structural characterization was performed with Project HOPE, and interaction networks were constructed using STRING and GeneMANIA. Results: Sequential filtering identified 37 high-risk missense variants. MuPro predicted destabilizing effects for 36/37 variants, with concordant DynaMut2 results for 35/37. Project HOPE identified disulfide bond disruption in 11 variants, charge-altering substitutions in 8, and glycine/proline backbone conformational changes in 11. STRING analysis identified IL12RB1 (0.999), IL23A (0.999), JAK2 (0.995), IL12B (0.986), and STAT3 (0.980) as the leading IL23R interactors. The protective variant R381Q was appropriately characterized as neutral by PROVEAN (−1.16) and AlphaMissense (likely_benign), supporting the specificity of the pipeline. Conclusions: Comprehensive in silico analysis identified 37 high-risk IL23R missense candidates with convergent computational evidence of predicted deleteriousness, predominantly involving cysteine bridge disruption, charge alteration, and glycine/proline backbone conformational changes. These variants are presented as prioritized candidates for future functional validation and may inform subsequent investigations of IBD susceptibility and IL-23 pathway pharmacogenomics.

## 1. Introduction

The interleukin-23 (IL-23)/T helper 17 (Th17) immune axis constitutes a central regulatory node in host defense against extracellular pathogens and in the pathogenesis of immune-mediated inflammatory diseases. IL-23 is a heterodimeric cytokine composed of a unique p19 subunit (encoded by *IL23A*) and a shared p40 subunit (encoded by *IL12B*, also shared with *IL-12*) [1,2]. Signaling through the IL-23 receptor complex drives the differentiation and maintenance of Th17 cells and promotes production of the downstream effector cytokines IL-17A, IL-17F, IL-22, and IFN-γ, which collectively orchestrate mucosal immunity and contribute to tissue inflammation in inflammatory bowel disease (IBD), psoriasis, psoriatic arthritis, Behcet’s disease, systemic lupus erythematosus, and ankylosing spondylitis [3,4,5,6,7,8,9,10,11,12,13,14].

The IL-23 receptor (IL-23R), encoded by *IL23R* on chromosome 1p31.3, forms a heterodimeric signaling complex with IL-12 receptor β1 (IL12RB1) upon IL-23 ligand engagement. Downstream signaling proceeds through JAK2 and TYK2 kinase activation, leading to STAT3 and STAT4 phosphorylation and subsequent transcriptional regulation of Th17 effector programs [3]. The clinical relevance of this pathway is underscored by the therapeutic success of biologics targeting key components, namely ustekinumab (anti-p40/IL12B), guselkumab, risankizumab, and tildrakizumab (anti-p19/IL23A), which are widely approved for IBD, psoriasis, and related conditions [15].

The *IL23R* was first identified as a susceptibility locus for Crohn’s disease in a landmark genome-wide association study (GWAS) [16]. Subsequently, numerous GWAS studies have confirmed associations between *IL23R* variants and IBD, psoriasis, and ankylosing spondylitis. The most studied variant, rs11209026 (R381Q; p.Arg381Gln), results in a glutamine substitution at a cytoplasmic arginine residue and confers substantial protection against IBD through reduced receptor signaling capacity [5]. Studies have shown that it promotes a soluble receptor isoform, reduces STAT3 phosphorylation, dampens Th17 responses, and results in reduced IL-23-induced signaling [5,17,18]. Three protective *IL23R* variants (V62I, I119N, and R381Q) have been characterized and shown to exhibit loss of function due to impaired protein stability, defective glycosylation, and disrupted intracellular trafficking to the cell surface [6]. Synthetic deletion of the IL23R stalk region has been shown to lead to autonomous receptor homodimerization and activation [19]. However, the complete landscape of *IL23R* missense variation has not been systematically characterized at the computational level.

In silico approaches for variant effect prediction have become indispensable for prioritizing functionally significant variants from the large and growing reservoir of catalogued human genetic variation [20]. Convergent predictions across multiple independent computational tools substantially reduce false discovery rates and provide mechanistic hypotheses for subsequent experimental validation. Tools employing evolutionary conservation (SIFT, PROVEAN), structural modeling (PolyPhen-2), deep learning (AlphaMissense), and functional impact inference (MutPred2) have each demonstrated independent predictive value, and their combination in sequential filtering pipelines has been validated in multiple recent studies of cytokine receptor genes [21,22,23,24,25].

A systematic in silico characterization of *IL23R* missense variants is particularly timely given the expanding pharmacogenomic relevance of this receptor. Genetic variation in *IL23R* may modulate inter-individual variability in biologic treatment response, and identification of high-risk variants could, in principle, contribute to future precision medicine investigations in biologic-treated IBD and psoriasis patients, although such applications remain to be established [26]. The present study applies a six-tool sequential filtering pipeline (SIFT, PolyPhen-2, PROVEAN, E-SNPs&GO, MutPred2, and ConSurf), complemented by two independent annotation layers (AlphaMissense and FATHMM-MKL), followed by comprehensive protein stability and structural analyses, to identify and characterize 37 high-risk missense variants in human *IL23R*.

## 2. Materials and Methods

### 2.1. Data Retrieval and Variant Filtering

Missense SNVs in *IL23R* (Gene ID: 149233) were retrieved from NCBI dbSNP (https://www.ncbi.nlm.nih.gov/snp/, accessed on 5 May 2026) [27]. After filtering for missense annotation and exclusion of synonymous co-annotations, 723 variants were retained. The UniProt canonical isoform Q5VWK5-1 (629 amino acids; transcript NM_144701.3, protein NP_653302.2) served as the reference sequence throughout [28]. ClinVar clinical significance annotations and gnomAD v4.1 allele frequencies were recorded for all 37 final high-risk variants and are summarized in Section 3.1 and provided as Supplementary Table S1 [29,30].

### 2.2. Variant Effect Prediction

Ensembl Variant Effect Predictor (VEP; GRCh38.p14, release 115) with RefSeq transcripts was used for annotation [31]. Results were filtered to transcript NM_144701.3 after positional verification against Q5VWK5-1, yielding 524 confirmed missense variants. SIFT (score < 0.05 = deleterious) and PolyPhen-2 (possibly/probably damaging) were applied; AlphaMissense classifications were obtained via VEP [21,23,24]. Variants predicted deleterious/damaging by both SIFT and PolyPhen-2 (*n* = 158) were advanced to PROVEAN (score ≤ −2.5 = deleterious; *n* = 65) [9]. AlphaMissense, a proteome-wide deep-learning model trained on evolutionary and structural features distinct from SIFT and PolyPhen-2, provided an independent pathogenicity classification layer and was not applied as a sequential exclusion criterion.

### 2.3. Pathogenicity and Functional Impact Prediction

E-SNPs&GO (https://esnpsandgo.biocomp.unibo.it/, accessed on 5 May 2026), a machine learning predictor using protein sequence embeddings, classified 44 of 65 variants as pathogenic [32]. FATHMM-MKL (http://fathmm.biocompute.org.uk/, accessed on 5 May 2026) was applied in weighted mode but predicted 47 of 65 variants as tolerated; given the known mode-dependent sensitivity of this tool for rare missense variants, it was used for annotation purposes only and not as a sequential exclusion criterion [33]. MutPred2 (http://mutpred.mutdb.org, accessed on 6 May 2026) was applied to the 44 pathogenic E-SNPs&GO variants; those scoring > 0.5 (*n* = 42) were retained [25].

### 2.4. Evolutionary Conservation

ConSurf was assessed for Q5VWK5-1 via Google Colab (https://consurf.tau.ac.il, accessed on 6 May 2026), using the Bayesian method, MAFFT alignment, and 150 homologous sequences [34]. Variants at positions with ConSurf grade ≥ 7 (*n* = 37) were designated high-risk and retained for downstream analyses.

### 2.5. Protein Stability Analysis

MuPro (https://mupro.proteomics.ics.uci.edu/, accessed on 6 May 2026) was applied to all 37 high-risk variants using the sequence-based SVM method; ΔΔG < 0 indicates destabilization [35]. DynaMut2 (https://biosig.lab.uq.edu.au/dynamut2/, accessed on 6 May 2026) was applied using the AlphaFold2 model AF-Q5VWK5-F1-v6 (pLDDT = 68.19) [36]. Per-residue pLDDT values at each of the 37 variant positions were extracted from the AlphaFold2 model and are provided in Supplementary Table S1.

### 2.6. Three-Dimensional Structural Analysis

Project HOPE was applied to all 37 variants using the Q5VWK5 UniProt accession (https://www3.cmbi.umcn.nl/hope/, accessed on 6 May 2026). Where available, structural information was obtained from PDB entry 5MZV; otherwise, the AlphaFold2 model was used [37]. The structural source used for each variant (PDB 5MZV or AlphaFold2 model) is indicated in Supplementary Table S1. Size, charge, hydrophobicity, cysteine bridge, hydrogen bond, and domain annotations were recorded for each variant.

### 2.7. Gene–Gene Interaction Network Analysis

Protein–protein interaction networks were constructed using STRING v12.0 (https://string-db.org, accessed on 6 May 2026) with *Homo sapiens* settings, a minimum interaction score of 0.700, and 10 first-shell interactors [38]. Complementary gene co-expression and functional association networks were generated using GeneMANIA (https://genemania.org, accessed on 6 May 2026) with 20 related genes [39].

## 3. Results

### 3.1. Variant Filtering Pipeline

**Figure 1:**
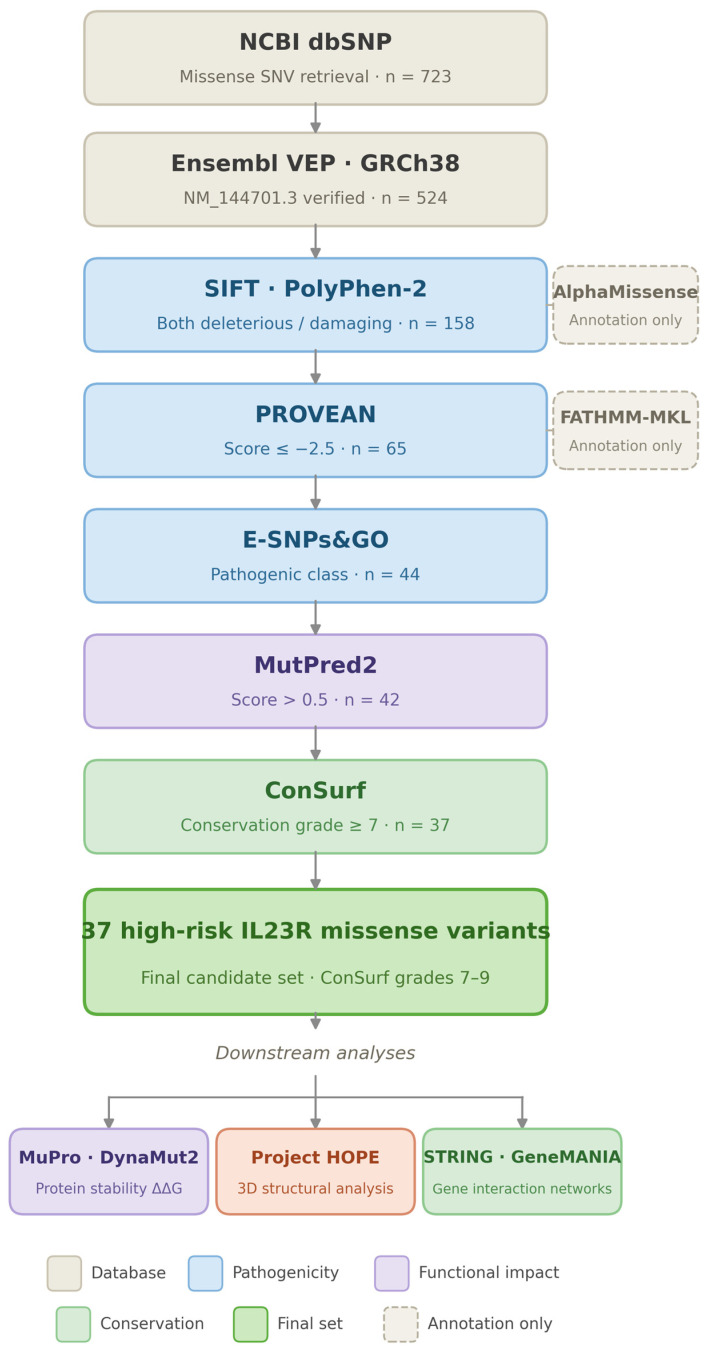
Sequential in silico filtering pipeline applied to *IL23R* missense single-nucleotide variants. Color coding: gray = database/retrieval steps; blue = pathogenicity prediction tools; purple = functional impact; green = evolutionary conservation and final high-risk variant set; dashed boxes = annotation-only tools (AlphaMissense, FATHMM-MKL).

Of 723 missense SNVs retrieved from NCBI dbSNP, 524 were confirmed at the Q5VWK5-1 reference sequence via VEP and assigned to transcript NM_144701.3. Sequential filtering through SIFT and PolyPhen-2 yielded 158 variants predicted deleterious/damaging by both tools, including the known protective variant R381Q (rs11209026; SIFT 0.02, PolyPhen-2 probably_damaging 0.995). PROVEAN filtering (score ≤ −2.5) reduced the set to 65 variants (R381Q: −1.16, neutral, appropriately excluded from subsequent filters). E-SNPs&GO identified 44 pathogenic variants; MutPred2 retained 42 (score > 0.5). ConSurf conservation filtering (grade ≥ 7) produced the final high-risk set of 37 variants. The complete pipeline is summarized in Figure 1. Population allele frequencies from gnomAD v4.1 and ClinVar clinical significance annotations for the 37 final variants are summarized in Supplementary Table S1. The majority of high-risk variants are extremely rare (gnomAD exomes AF < 10^−4^) or not reported in gnomAD, consistent with their predicted deleteriousness. Seven variants carried ClinVar uncertain significance annotations; one variant, G149R (rs76418789), carried a ClinVar benign classification and an elevated gnomAD exomes allele frequency (6.28 × 10^−3^). Although it passed all computational filters, it was excluded from the final candidate list based on this population-level and clinical annotation evidence, consistent with prior experimental characterization as a loss-of-function protective variant [6].

FATHMM-MKL predicted 18 of 65 PROVEAN-filtered variants as damaging and 47 as tolerated. Given this reduced sensitivity in weighted mode, FATHMM-MKL results were retained as an annotation layer rather than used as a hard filter.

Population allele frequency and ClinVar annotation data were retrieved through Ensembl VEP (GRCh38.p14, release 115) by querying gnomAD v4.1 databases. Of the 37 high-risk variants, 24 had reportable gnomAD v4.1 exomes allele frequencies (global AF), all below 1 × 10^−4^; the highest was V160A (rs201902670; 7.1 × 10^−5^), with the majority falling below 1 × 10^−5^, consistent with their predicted deleteriousness. Thirteen variants were absent from the gnomAD v4.1 exomes database, indicating extreme rarity in currently catalogued populations. Seven variants carried ClinVar uncertain significance (VUS) annotations. The remaining 30 variants had no ClinVar entry, reflecting their ultra-rare population frequency.

### 3.2. Functional Impact and Pathogenicity Predictions

Among the 37 high-risk variants, all were predicted deleterious by SIFT (scores 0.00–0.04) and damaging by PolyPhen-2 (scores 0.486–1.000). AlphaMissense classified 28 variants as likely pathogenic and 8 as ambiguous. PROVEAN scores ranged from −2.515 to −11.311, with the most extreme values observed for W265C (−11.311), W265S (−11.228), and W307G (−10.853). E-SNPs&GO pathogenicity probabilities ranged from 0.515 to 0.933; the highest were assigned to C101W and C296R (both 0.933), with C115R, C253R, L202P, W307G, and S308R also exceeding 0.930. MutPred2 scores ranged from 0.522 to 0.940; 14 variants exceeded 0.8, led by C296R (0.940), C52F (0.913), and W307G (0.902).

### 3.3. Protein Stability Analysis

MuPro predicted destabilizing effects (ΔΔG < 0) for 36 of 37 variants, ranging from −0.096 to −2.389 kcal/mol. The strongest MuPro destabilization was predicted for L202P (−2.389), V218G (−2.290), C115G (−2.206), G116A (−1.811), and C115S (−1.733 kcal/mol). W195L was the only variant predicted to increase stability by MuPro (+0.198 kcal/mol). DynaMut2 corroborated MuPro for 35 of 37 variants; W395R was the sole additional discordant variant (MuPro: decrease; DynaMut2: +0.33 kcal/mol). Ten showed concordant strong destabilization (ΔΔG < −1.0 kcal/mol by both tools), including W307G (DynaMut2: −3.10 kcal/mol), V160A (DynaMut2: −2.45 kcal/mol), W265S (DynaMut2: −2.27 kcal/mol), and V218G (DynaMut2: −1.97 kcal/mol). Complete stability results are provided in Table 1.

### 3.4. Three-Dimensional Structural Characterization

Structural analysis using Project HOPE predicted four recurrent mechanisms through which these substitutions could destabilize the protein. First, disulfide bond disruption was predicted for 11 variants (C52F, C59R, C101W, C101Y, C105W, C115R, C115G, C115Y, C115S, C253R, and C296R), representing 30% of the high-risk set. Seven of these cysteine-disrupting variants were located in the buried core of their respective domains, where disulfide bonds provide critical conformational rigidity. Second, charge-altering substitutions were identified in 8 variants: C59R, C115R, C253R, C296R, S308R, W395R, G483R (neutral-to-positive), and E254G (negative-to-neutral). The introduction of positively charged arginine residues at multiple positions throughout the extracellular domain would be expected to create charge repulsion with surrounding residues and perturb receptor complex assembly. Third, backbone conformational constraints involving glycine and proline residues were identified in 11 variants. Six substitutions remove a native glycine (G116A, G149V, G203A, G203S, G483V, and G483R): because glycine is the only residue able to access the full range of backbone torsion angles, replacing it forces the local backbone into a conformation incompatible with the native geometry. One substitution instead introduces a glycine (V218G), conferring excess backbone flexibility at a position that requires conformational rigidity. The remaining four involve proline (L202P, P220S, P220T, and P401S): L202P introduces a conformationally rigid proline into the backbone, whereas P220S, P220T, and P401S abolish a native proline and the rigidity it provides. Fourth, hydrogen bond losses were predicted for multiple variants, including W35C (loss of contact with Glu37), C101Y, C115Y, C115S, W195C, W265C, W265S, Y290F, and W307G. Full structural characterization is provided in Table 2. Five variants spanning the principal destabilization mechanisms and the highest convergent scores (C52F, C59R, L202P, C296R, and W307G) were selected for detailed three-dimensional visualization (Table 3).

### 3.5. Gene–Gene Interaction Network Analysis

**Figure 2:**
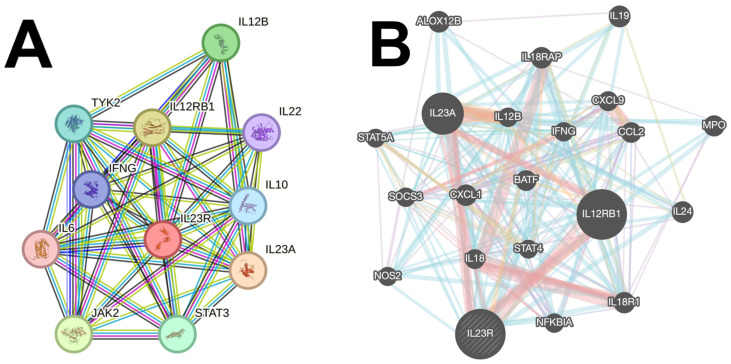
Protein–protein interaction and gene co-expression networks of IL23R. (**A**) STRING v12.0 protein–protein interaction network of IL23R (*Homo sapiens*). The network was constructed using a minimum combined interaction score threshold of 0.700 with 10 first-shell interactors. IL23R (central node) is displayed alongside its ten highest-confidence interactors. Edge colors represent distinct evidence channels: green = gene neighborhood; red = gene fusion; blue = phylogenetic co-occurrence; black = co-expression; pink (magenta) = experimentally determined interactions; yellow = text-mining; light blue = curated biological databases. Node size reflects the interaction degree. The five strongest direct IL23R interactions are IL12RB1 (combined score 0.999), IL23A (0.999), JAK2 (0.995), IL12B (0.986), and STAT3 (0.980). (**B**) GeneMANIA functional gene interaction network of *IL23R*. The network displays *IL23R* (central hatched node) and 20 functionally related genes identified through co-expression, predicted interaction, physical interaction, co-localization, genetic interaction, and pathway-based evidence. Node size reflects relative network weight. Edge thickness corresponds to interaction confidence. Key network members include IL12RB1, IL23A, IL12B, and IFNG (shared with the STRING network), together with GeneMANIA-specific associations including *NFKBIA*, *SOCS3*, *BATF*, *STAT4*, *CXCL1*, *CCL2*, *IL18*, and *NOS2*, collectively providing network-level context for IL23R involvement in NF-κB regulation, Th17 differentiation, and IBD-associated inflammatory signaling. Edge colors denote the evidence type: salmon/pink = physical interactions; purple = co-expression; orange = predicted; blue = co-localization; green = genetic interactions; light blue = pathway; gold = shared protein domains.

STRING network analysis of IL23R with *Homo sapiens* settings (minimum confidence 0.700) produced a network of 11 nodes and 50 edges. The strongest direct interactions with IL23R were IL12RB1 (combined score 0.999), IL23A (0.999), JAK2 (0.995), IL12B (0.986), STAT3 (0.980), TYK2 (0.979), IL10 (0.970), IFNG (0.959), IL22 (0.947), and IL6 (0.934) (Figure 2A). The IL12RB1-IL23R interaction was supported by both experimental evidence and database annotation, consistent with their established role as obligate heterodimeric receptor components. The IL23A-IL23R interaction was likewise experimentally confirmed, reflecting direct ligand–receptor binding.

GeneMANIA network analysis revealed 20 functionally related genes to *IL23R*, including *IL12RB1* and *IL23A* as the highest-weighted co-expressed and co-localized genes. Additional network members not represented in the STRING analysis included *NFκBIA* (NF-kB inhibitor alpha), *SOCS3* (suppressor of cytokine signaling 3), *BATF* (basic leucine zipper ATF-like transcription factor), *STAT4*, *STAT5A*, *CXCL1*, *CCL2*, *IL18*, *IL18R1*, *IL18RAP*, *IL19*, *IL24*, *NOS2*, *CXCL9*, *ALOX12B*, and *MPO*. The presence of *NFKBIA* and *SOCS3* in the GeneMANIA network highlights the importance of IL23R in NF-kB pathway regulation and negative feedback control of cytokine signaling. BATF and STAT4 connections reinforce the role of IL23R in Th17 and Th1 cell differentiation programs relevant to IBD pathogenesis (Figure 2B).

## 4. Discussion

This study presents a systematic multi-tool in silico analysis of the full *IL23R* missense variant landscape, identifying 37 high-risk candidates through convergent computational evidence. The pipeline spans six sequential filtering tools and two independent annotation layers, two stability analyses, comprehensive structural characterization, and gene network analysis, providing a robust prioritization framework grounded in orthogonal evidence types, from evolutionary conservation to deep-learning-based functional impact inference. This is, to date, the first whole-landscape in silico characterization of IL23R missense variation (723 variants) to integrate mechanism-based structural classification with an internal specificity check using the protective variant R381Q. The principal contribution is therefore a structurally annotated, reproducible candidate set for downstream functional study rather than a new prediction algorithm.

The predominance of cysteine-disrupting variants (11 of 37; 30%) among the high-risk set is mechanistically coherent. Disulfide bonds are among the most critical stabilizing elements in cytokine receptor extracellular domains, maintaining the three-dimensional architecture required for ligand recognition and receptor complex assembly [40]. The IL23R extracellular domain contains multiple cysteine residues essential for domain folding; their mutation to non-cysteine amino acids, particularly to bulky aromatic residues (phenylalanine, tryptophan, tyrosine) or charged residues (arginine), would eliminate the disulfide bond and introduce steric or electrostatic perturbations simultaneously. C52F and C59R, both located in the buried core where disulfide bonds are structurally critical, represent particularly high-priority candidates for experimental validation.

The eight charge-altering variants identified by Project HOPE represent an independent destabilization mechanism. Among these, seven introduce positively charged arginine residues (C59R, C115R, C253R, C296R, S308R, W395R, and G483R) at positions where the native residue is neutral. Introduction of a charged residue into a hydrophobic core or at a domain interface typically disrupts packing interactions and creates electrostatic incompatibilities with surrounding residues [41]. C296R, which achieved the highest MutPred2 score (0.940) in the dataset, combines cysteine bridge disruption, charge introduction, and predicted strong destabilization (MuPro −1.290, DynaMut2 −1.080 kcal/mol), making it the highest-priority candidate for functional disruption among the variants analyzed, based on convergent computational evidence. Notably, position Cys115 recurs four times in the high-risk set (C115R, C115G, C115Y, and C115S), each predicted to disrupt the same conserved disulfide-forming cysteine. This clustering identifies Cys115 as a likely structural mutational hotspot in the IL-23R extracellular domain and a priority site for experimental characterization.

The glycine/proline backbone conformational-change category (11 variants) comprises three distinct mechanisms, which are glycine loss, glycine gain, and proline alteration. Among the glycine-loss substitutions, the effect is particularly consequential because glycine is the sole residue able to access torsion angles inaccessible to all other amino acids; its replacement forces the local backbone into a conformation incompatible with the native protein geometry, destabilizing secondary structure and potentially disrupting long-range structural contacts [42]. The G116A, G149V, G203A/S, and G483V/R substitutions, all at highly conserved positions (ConSurf grade ≥ 7), would be expected to propagate backbone distortions beyond the local mutation site.

The behavior of the known protective variant R381Q (rs11209026) provides an important internal consistency check for the pipeline. R381Q was retained through SIFT and PolyPhen-2 filtering (SIFT 0.02, PolyPhen-2 0.995) but was correctly excluded by PROVEAN (score −1.16, neutral). AlphaMissense classified it as likely_benign (score 0.137), consistent with its established role as a reduced- or altered-function variant rather than a classical deleterious missense mutation causing receptor loss. This behavior is biologically appropriate: R381Q reduces JAK2 association and STAT3 activation but does not abolish receptor expression or trafficking [5]. The pipeline’s ability to correctly characterize R381Q as neutral supports its internal consistency and specificity. It is emphasized that correct classification of a single benchmark variant cannot establish the overall predictive performance of the pipeline; R381Q provides an internal specificity check rather than a validation of sensitivity, which would require a curated gold-standard set of functionally characterized IL23R variants.

FATHMM-MKL classified only 18 of 65 PROVEAN-filtered variants as damaging, with the remaining 47 predicted as tolerated. This discordance is consistent with the known behavior of FATHMM-MKL’s inherited-disease weighted mode, whose sensitivity for rare missense variants depends strongly on the density of pathogenic training annotations available for a given gene family [33]. Because variant-effect predictors differ substantially in sensitivity and are best interpreted in combination rather than in isolation, the FATHMM-MKL output was retained as a supplementary annotation layer rather than used as a hard filter, and its tolerated calls should not be taken as evidence against variant pathogenicity [20].

STRING network analysis identified IL12RB1 and IL23A as the highest-confidence direct interactors of IL23R (both scores 0.999), reflecting the obligate heterodimeric receptor complex architecture and the specific ligand–receptor relationship that are well-established biochemically [43]. The strong interactions with JAK2 (0.995) and STAT3 (0.980) confirm the canonical downstream signaling pathway through which IL23R variants would exert their phenotypic effects [3]. Importantly, variants affecting the extracellular domain, as most of the cysteine-disrupting variants do, could perturb IL23A or IL12RB1 binding geometry and thereby impair JAK-STAT activation even without direct involvement of the cytoplasmic JAK2-binding region. GeneMANIA network data further contextualize the IBD relevance: IκBα and SOCS3 are canonical negative regulators of cytokine signaling whose network connections to IL23R suggest that IL23R variant effects would propagate through NFκB and JAK-STAT feedback circuits central to IBD pathogenesis.

The detected variants may also carry pharmacogenomic relevance, although this possibility remains speculative and currently untested. Existing IL-23-pathway biologics act at distinct points of the axis: risankizumab and guselkumab target the IL-23A (p19) subunit, ustekinumab targets the shared p40 subunit (IL12B), and brodalumab targets the downstream IL-17RA receptor [15]. Because these agents engage the ligand or downstream effectors rather than IL-23R itself, any influence of IL23R coding variants on treatment response would most plausibly be indirect (i.e., through altered receptor-complex assembly) and no such effect has yet been demonstrated. Whether the high-risk variants prioritized here modulate biologic efficacy therefore remains an open question that will require dedicated pharmacogenomic studies in treated patient cohorts.

The IL23R locus has been robustly replicated as a susceptibility gene across the full spectrum of IBD. Following the initial discovery in Crohn’s disease, subsequent GWAS and meta-analyses have extended *IL23R* associations to ulcerative colitis, establishing the locus as a shared genetic risk factor for both major IBD subtypes [16,44,45]. The Wellcome Trust Case Control Consortium identified *IL23R* among the leading susceptibility loci in a landmark multi-disease analysis, and subsequent fine-mapping studies have implicated multiple independent signals within the locus [46,47,48,49]. High-risk missense variants that severely impair IL-23R extracellular domain integrity, as predicted for the cysteine-disrupting variants in the current study, could, if functionally confirmed, represent rare alleles that contribute to IBD risk by perturbing the regulatory balance between pro-inflammatory IL-23 signaling and immune tolerance at mucosal surfaces. Such variants would be expected to skew Th17 differentiation and promote sustained intestinal epithelial barrier disruption, the two hallmark pathomechanisms of IBD [50].

Beyond IBD, *IL23R* missense variation has direct relevance to psoriasis and psoriatic arthritis. GWASes in European populations have identified IL23R as a major susceptibility locus for plaque psoriasis, with multiple non-synonymous variants at this locus conferring altered risk [9,51]. The IL-23/Th17 axis drives keratinocyte hyperproliferation and sustained neutrophilic skin inflammation in psoriatic lesions through IL-17A-dependent mechanisms [52]. High-risk missense variants affecting the IL-23R extracellular domain could theoretically modulate the threshold for IL-23-driven Th17 activation in skin, altering individual susceptibility to psoriasis development or influencing the balance between cutaneous and intestinal disease manifestations. Ankylosing spondylitis represents a third inflammatory condition in which *IL23R* variants have been reproducibly identified as susceptibility determinants, implicating this receptor in entheseal inflammation and axial spondyloarthropathy pathogenesis [53]. The high-risk *IL23R* variants prioritized here may be relevant across the spondyloarthritis–IBD–psoriasis disease spectrum, although this relevance remains hypothetical pending experimental validation.

Several limitations should be acknowledged. First, all findings are derived from computational prediction and are therefore hypothesis-generating; none of the prioritized variants has been experimentally validated, and functional confirmation in cellular or biochemical assays will be required before clinical inferences can be drawn. Second, the prediction tools are not fully independent. SIFT and PROVEAN both rely heavily on evolutionary conservation, and several methods share overlapping training data, so convergent predictions reduce but do not eliminate correlated error. Third, the structural analyses based on the AlphaFold2 model (AF-Q5VWK5-F1-v6) should be interpreted with caution, as the model carries only moderate per-residue confidence (mean pLDDT = 68.19), and the experimental structure 5MZV resolves only part of the receptor; predicted destabilization magnitudes and interaction-map changes are therefore tentative. However, the variant positions themselves are predominantly well modeled: 33 of the 37 show per-residue pLDDT > 85 (Supplementary Table S1), and only W395R, P401S, and the G483 variants fall below 70 (C-terminal/cytoplasmic region); the lower global mean reflects disordered segments elsewhere in the receptor. Predictions for these three positions should therefore be treated with particular caution, whereas the high-priority extracellular cysteine candidates lie in well-modeled regions. Finally, population-frequency and ClinVar annotations reflect currently cataloged variation and may change as databases expand. These limitations notwithstanding, the convergent multi-tool framework provides a prioritized, experimentally testable candidate list rather than definitive functional conclusions.

## 5. Conclusions

In conclusion, this study provides a systematic in silico characterization of missense SNVs in the human *IL23R* gene, identifying 37 high-risk candidates through convergent evidence from six complementary computational tools and two independent annotation layers. The predominance of cysteine bridge-disrupting variants (11 of 37), charge-altering substitutions (8), and glycine/proline backbone conformational changes (11) provides a structurally coherent picture of the mechanisms through which *IL23R* missense variation could impair receptor function. The strong interaction network centered on IL12RB1, IL23A, JAK2, and STAT3 places these variants in the context of the IL-23/Th17 signaling cascade critically implicated in IBD pathogenesis. C296R, C52F, C59R, W307G, and L202P emerge as the highest-priority candidates for future experimental functional validation. Any implications for IBD genetic susceptibility or IL-23 pathway pharmacogenomics remain to be established and should be regarded as hypotheses for subsequent functional and clinical studies.

## Figures and Tables

**Table 1:** Protein stability analysis of 37 high-risk *IL23R* missense variants using MuPro and DynaMut2 ^1^.

Variant	MuPro ΔΔG (kcal/mol)	MuPro Pred.	DynaMut2 ΔΔG (kcal/mol)	DynaMut2 Pred.	Concordance
W35C	−0.628	Decrease	−0.43	Decrease	✓
C52F	−0.838	Decrease	−1.22	Decrease	✓
C59R	−0.887	Decrease	−0.85	Decrease	✓
C101W	−1.109	Decrease	−1.26	Decrease	✓✓
C101Y	−0.947	Decrease	−0.73	Decrease	✓
C105W	−1.052	Decrease	−1.05	Decrease	✓✓
C115R	−1.311	Decrease	−0.81	Decrease	✓
C115G	−2.206	Decrease	−0.94	Decrease	✓
C115Y	−1.168	Decrease	−0.55	Decrease	✓
C115S	−1.733	Decrease	−1.27	Decrease	✓✓
G116A	−1.811	Decrease	−0.58	Decrease	✓
P124L	−0.096	Decrease	−0.39	Decrease	✓
G149V	−1.164	Decrease	−1.24	Decrease	✓✓
V160A	−1.719	Decrease	−2.45	Decrease	✓✓
W195C	−0.331	Decrease	−0.29	Decrease	✓
W195L	+0.198	Increase	−2.98	Decrease	✗
V196L	−0.173	Decrease	−0.41	Decrease	✓
L202P	−2.389	Decrease	−0.42	Decrease	✓
G203A	−1.246	Decrease	−0.39	Decrease	✓
G203S	−0.985	Decrease	−0.34	Decrease	✓
V218G	−2.290	Decrease	−1.97	Decrease	✓✓
P220S	−0.694	Decrease	−0.51	Decrease	✓
P220T	−0.837	Decrease	−0.68	Decrease	✓
W242C	−0.967	Decrease	−0.88	Decrease	✓
C253R	−0.687	Decrease	−0.74	Decrease	✓
E254G	−1.605	Decrease	−0.30	Decrease	✓
W265C	−1.025	Decrease	−0.13	Decrease	✓
W265S	−1.540	Decrease	−2.27	Decrease	✓✓
L284W	−1.168	Decrease	−1.74	Decrease	✓✓
Y290F	−0.691	Decrease	−0.41	Decrease	✓
C296R	−1.290	Decrease	−1.08	Decrease	✓✓
W307G	−1.086	Decrease	−3.10	Decrease	✓✓
S308R	−0.846	Decrease	−0.67	Decrease	✓
W395R	−1.167	Decrease	+0.33	Increase	✗
P401S	−1.341	Decrease	−0.18	Decrease	✓
G483V	−0.703	Decrease	−1.15	Decrease	✓
G483R	−1.025	Decrease	−0.41	Decrease	✓

^1^ ΔΔG < 0 = destabilizing; ΔΔG > 0 = stabilizing (kcal/mol). ✓ = concordant prediction (both tools agree on the direction of the stability change). ✓✓ = concordant strong destabilization (<−1.0 kcal/mol by both tools). ✗ = discordant prediction.

**Table 2:** Three-dimensional structural characterization of 37 high-risk *IL23R* missense variants using Project HOPE ^1^.

Variant	Size	Charge	Hydrophobicity	Disulfide Bond	Structural Effect	Location
W35C	Mutant smaller	No change	Changed	−	H-bond loss (Glu37)	Surface
C52F	Mutant larger	No change	Changed	Disrupted ‡	Steric clash	Buried core
C59R	Mutant larger	Neutral → Positive	Loss	Disrupted ‡	Charge introduction	Buried core
C101W	Mutant larger	No change	Changed	Disrupted ‡	Steric clash	Buried core
C101Y	Mutant larger	No change	Loss	Disrupted ‡	H-bond loss	Buried core
C105W	Mutant larger	No change	Changed	Disrupted ‡	Steric clash	Buried core
C115R	Mutant larger	Neutral → Positive	Loss	Disrupted ‡	Charge introduction	Surface
C115G	Mutant smaller	No change	Loss	Disrupted ‡	Flexibility gain	Surface
C115Y	Mutant larger	No change	Loss	Disrupted ‡	H-bond loss	Surface
C115S	No change	No change	Loss	Disrupted ‡	H-bond loss	Surface
G116A	Mutant larger	No change	More hydrophobic	−	Flexibility loss	Surface
P124L	Mutant larger	No change	Changed	−	Proline rigidity loss	Surface
G149V	Mutant larger	No change	More hydrophobic	−	Flexibility loss	Surface
V160A	Mutant smaller	No change	Changed	−	Core destabilization	−
W195C	Mutant smaller	No change	Changed	−	H-bond loss	−
W195L	Mutant smaller	No change	Changed	−	Hydrophobic change	−
V196L	Mutant larger	No change	Changed	−	Core destabilization	Buried core
L202P	Mutant smaller	No change	Changed	−	Proline introduction	−
G203A	Mutant larger	No change	More hydrophobic	−	Flexibility loss	Surface
G203S	Mutant larger	No change	Changed	−	Flexibility loss	Surface
V218G	Mutant smaller	No change	Loss	−	Flexibility gain	−
P220S	Mutant smaller	No change	Loss	−	Proline rigidity loss	−
P220T	No change	No change	Loss	−	Proline rigidity loss	−
W242C	Mutant smaller	No change	Changed	−	H-bond loss	−
C253R	Mutant larger	Neutral → Positive	Loss	Disrupted ‡	Charge + disulfide	Buried core
E254G	Mutant smaller	Negative → Neutral	More hydrophobic	−	Charge loss	−
W265C	Mutant smaller	No change	Changed	−	H-bond loss	−
W265S	Mutant smaller	No change	Loss	−	H-bond loss	−
L284W	Mutant larger	No change	Changed	−	Steric clash	Buried core
Y290F	Mutant smaller	No change	More hydrophobic	−	H-bond loss	−
C296R	Mutant larger	Neutral → Positive	Loss	Disrupted ‡	Charge + disulfide	Buried core
W307G	Mutant smaller	No change	Loss	−	H-bond loss	−
S308R	Mutant larger	Neutral → Positive	Loss	−	Charge introduction	Buried core
W395R	Mutant smaller	Neutral → Positive	Loss	−	Charge introduction	−
P401S	Mutant smaller	No change	Loss	−	Proline rigidity loss	−
G483V	Mutant larger	No change	More hydrophobic	−	Flexibility loss	−
G483R	Mutant larger	Neutral → Positive	Loss	−	Charge introduction	−

^1^ ‡ = annotated disulfide bond disrupted. A dash (–) in the Location column indicates positions for which a buried-core versus surface assignment could not be unambiguously determined from the available structural model (residues outside the region resolved in PDB 5MZV or within lower-confidence AlphaFold2 segments). All variants were analyzed using PDB 5MZV, where available; otherwise, the AlphaFold2 model AF-Q5VWK5-F1-v6 was used. Amino acid exchange diagram showing the schematic structures of the wild-type (left) and mutant (right) amino acids, with the arrow indicating the wild-type → mutant substitution.

**Table 3:** Three-dimensional structural visualization of five representative high-risk *IL23R* missense variants using Project HOPE and DynaMut2 ^1^.

Variant Information	Project Hope		DynaMut2	
AA Change	Ribbon Model of Mutated Protein	Detailed View of Mutation	3D Interaction Map-WT	3D Interaction Map-Mutated
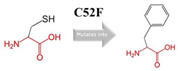	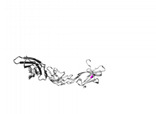	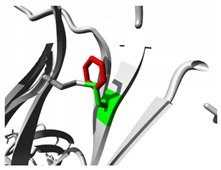	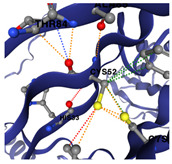	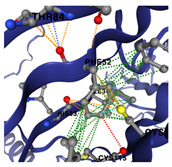
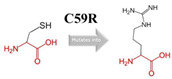	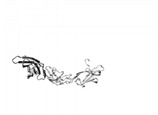	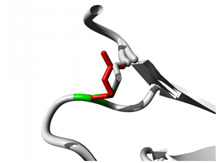	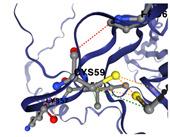	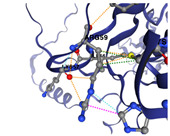
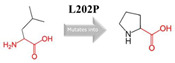	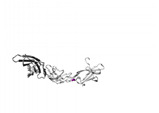	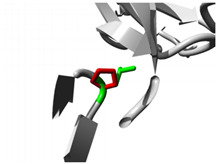	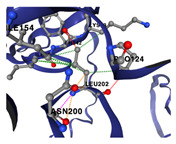	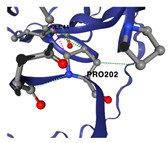
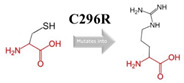	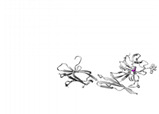	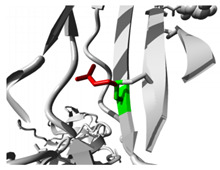	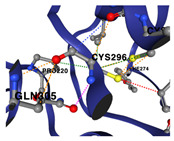	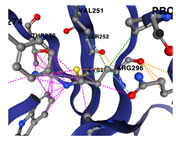
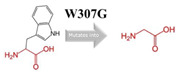	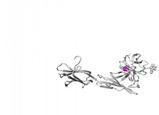	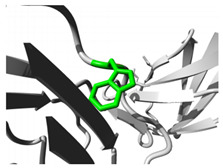	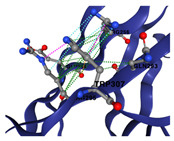	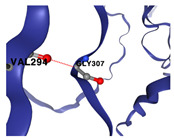

^1^ For each variant, five panels are presented from left to right: (1) Amino acid exchange diagram showing the schematic structures of the wild-type (left) and mutant (right) amino acids; the peptide backbone is shown in red and the side chain in black. (2) Full ribbon representation of the IL-23R protein model generated by Project HOPE using PDB 5MZV (where structurally resolved) or AlphaFold2 model AF-Q5VWK5-F1-v6, with the mutated residue indicated in magenta. (3) Close-up ribbon view of the substitution site, with the wild-type residue shown in green and the mutant residue in red. (4–5) DynaMut2 three-dimensional interaction maps of the wild-type (panel 4) and mutant (panel 5) residue environments, showing hydrogen bonds (red dashed lines), hydrophobic contacts (green dashed lines), ionic interactions (yellow dashed lines), carbonyl interactions (dark blue dashed lines), polar interactions (orange dashed lines), and van der Waals interactions (light blue dashed lines).

## Data Availability

The data supporting the results of this study are derived entirely from publicly accessible databases and web-based computational tools. IL23R missense single-nucleotide variants were retrieved from NCBI dbSNP (https://www.ncbi.nlm.nih.gov/snp/, accessed on 5 May 2026). Variant annotation, population allele frequency data (gnomAD v4.1), and ClinVar clinical significance annotations were obtained through Ensembl Variant Effect Predictor (VEP, GRCh38.p14, release 115; https://www.ensembl.org/Tools/VEP, accessed on 5 May 2026). Evolutionary conservation scores were computed using ConSurf (https://consurf.tau.ac.il, accessed on 6 May 2026) via a publicly available Google Colab notebook. All additional bioinformatics tools and databases used in this study are listed with their URLs in Section 2 and are freely accessible. No new experimental data were generated. The intermediate computational datasets generated during this study, including the filtered variant lists and annotated output files, are available from the corresponding author upon reasonable request.

## Supplementary Materials

The following supporting information can be downloaded at: https://www.mdpi.com/article/10.3390/genes17060699/s1, Table S1: Complete annotation of the 37 high-risk IL23R missense variants (all prediction-tool scores, gnomAD v4.1 allele frequencies, ClinVar significance, per-residue pLDDT, and structural source).

## Abbreviations

The following abbreviations are used in this manuscript:

AF	Allele frequency
GWAS	Genome-wide association study
IBD	Inflammatory bowel disease
IL	Interleukin
SNP	Single-nucleotide polymorphism
SNV	Single-nucleotide variant
VEP	Variant-effect predictor
VUS	Variant of uncertain significance
